# A randomized controlled trial to assess the efficacy of Parenting-STAIR in treating maternal PTSD to reduce maltreatment recidivism: protocol for the Safe Mothers, Safe Children study

**DOI:** 10.1186/s13063-022-06354-1

**Published:** 2022-05-23

**Authors:** Michael Lindsey, Kathrine Sullivan, Claude Chemtob, Kelly Ancharski, James Jaccard, Marylène Cloitre, Anthony Urquiza, Susan Timmer, Mercedes Okosi, Debra Kaplan

**Affiliations:** 1 Silver School of Social Work, New York University, New York, NY USA; 2 McSilver Institute for Poverty Policy and Research, Silver School of Social Work, New York University, New York, NY USA; 3 Grossman School of Medicine, New York University, New York, NY USA; 4 Institute for Trauma and Stress, New York University Langone Medical Center, New York, NY USA; 5 National Center for PTSD Dissemination and Training Division, Department of Psychiatry and Behavioral Sciences, Stanford University, Stanford, CA USA; 6 CAARE Diagnostic & Treatment Center, Department of Pediatrics, University of California, Sacramento, CA USA

**Keywords:** PTSD, Depression, Maltreatment recidivism, PCIT, STAIR, Supportive counseling, Randomized controlled trial

## Abstract

**Background:**

Child maltreatment recidivism substantially increases the likelihood of adverse life outcomes, but there is little evidence that family preservation services are effective at reducing recidivism. Mothers in child welfare have very high rates of trauma exposure; maternal post-traumatic stress disorder (PTSD) is an intervention target that has the potential to reduce abuse and neglect. The Safe Mothers, Safe Children (SMSC) intervention program involves the delivery of an innovative combination of interventions, including Skills Training in Affective and Interpersonal Regulation (STAIR) and Parent-Child Interaction Therapy (PCIT). The combined intervention, Parenting-STAIR (P-STAIR), targets maternal PTSD and comorbid depression symptoms to reduce the adverse effects of PTSD on parenting, improve positive parenting skills, and prevent maltreatment recidivism.

**Methods:**

This study is a two-arm randomized controlled trial: P-STAIR (23 sessions) versus supportive counseling (23 sessions). Participants are mothers receiving child welfare family preservation services (FPS), with a child in the age range of 1–8 years old and meeting diagnostic criteria for PTSD (with/without depression). Clinical assessment occurs at pre-treatment (baseline), two in-treatment assessments (mid-assessment #1 after module 9 and mid-assessment #2 after module 15), post-treatment, and at a 6-month follow-up. Recidivism will be measured using the New York State Child Welfare Registry (NYSCWR). We will enroll a total of 220 participants over 4 years: half (*N* = 110) randomly assigned to the P-STAIR condition and half (*N* = 110) to the supportive counseling condition.

**Discussion:**

This is the first RCT to investigate the efficacy of P-STAIR. The findings for the trial have the potential to contribute to the expansion of evidence-based practices for maternal PTSD, maltreatment, and child welfare.

## Administrative information

Note: the numbers in curly brackets in this protocol refer to SPIRIT checklist item numbers. The order of the items has been modified to group similar items (see http://www.equator-network.org/reporting-guidelines/spirit-2013-statement-defining-standard-protocol-items-for-clinical-trials/).

**Table:** 

Title {1}	A randomized controlled trial to assess the efficacy of Parenting-STAIR in treating maternal PTSD to reduce maltreatment recidivism: Protocol for the Safe Mothers, Safe Children study
Trial registration {2a and 2b}	ClinicalTrials.gov; ID: NCT04752618; February 2021
Protocol version {3}	V 1.1; July 20, 2021
Funding {4}	Study sponsored by The Eunice Kennedy Shriver National Institute of Child Health and Human Development (NICHD)
Author details {5a}	Michael Lindsey, PhD, MSW, MPH,^1,2^ Kathrine Sullivan, PhD, MSW,^1^ Claude Chemtob, PhD (deceased author),^1,3^ Kelly Ancharski, MSW,^2^ James Jaccard, PhD,^1^ Marylène Cloitre, PhD,^4,5^ Anthony Urquiza, PhD,^6^ Susan Timmer, PhD,^6^ Mercedes Okosi, PsyD,^2^ Debra Kaplan, PhD^2^ ^1^Silver School of Social Work, New York University, New York, New York ^2^McSilver Institute for Poverty Policy and Research, Silver School of Social Work, New York University, New York, New York ^3^Grossman School of Medicine, New York University, New York, New York ^4^Institute for Trauma and Stress, New York University Langone Medical Center, New York, New York ^5^National Center for PTSD Dissemination and Training Division, Department of Psychiatry and Behavioral Sciences, Stanford University, Stanford, California ^6^CAARE Diagnostic & Treatment Center, Department of Pediatrics, University of California, Sacramento, California
Name and contact information for trial sponsor {5b}	Michael Lindsey, PhD, MSW, MPH McSilver Institute for Poverty Policy and Research Telephone: (212) 998-5927 E-mail: ml4361@nyu.edu
Role of sponsor {5c}	The study sponsor has had no role in study design, collection, management, analysis, interpretation of data, report writing, or the decision to submit the protocol for publication.

## Introduction

### Background and rationale {6a}

Child maltreatment recidivism substantially increases the likelihood of adverse life outcomes [1–4]. The outcomes of repeated maltreatment include injury and disability, negative effects on brain and physical development, lifespan mental and physical health problems, greater suicide risk, and poor adaptation in young adulthood, including higher crime and incarceration rates [5]. Overall, the economic cost of maltreatment exceeds $124 billion annually [6]. There is little evidence that child welfare (CW) family preservation services (FPS) reduce maltreatment recidivism [7]. Families identified by CW as abusing or neglecting their children, whose children do not require immediate removal to foster care, are enrolled in family preservation programs to reduce the risk of re-abuse or neglect. FPS typically include safety monitoring, case management, crisis intervention, and parenting classes. FPS programs seek to preserve families, while ensuring child safety. Maltreatment reoccurrence rates as high as 69% have been reported even after receipt of FPS [8]. Given this, new approaches to reduce recidivism of child maltreatment are required.

Maternal post-traumatic stress disorder (PTSD) is an intervention target that has the potential to reduce abuse and neglect [9–13]. Prior work demonstrates that mothers receiving FPS to prevent recidivism have a high prevalence of trauma-related disorders: 54.3% met criteria for probable PTSD and 61.7% for depression [14]. High rates of PTSD among these mothers suggest that treating PTSD may reduce recidivism in a significant subset of high-risk mothers. In addition to directly increasing risk of maltreatment, maternal PTSD-related cognitive deficits may make learning parenting skills more difficult, contributing to the intergenerational persistence of trauma [15].

Between 2015 and 2020, the Safe Mothers, Safe Children (SMSC) intervention program conducted a single-arm pilot study to evaluate the feasibility and acceptability of Parenting-STAIR (P-STAIR). Pilot data (*N* = 78) indicates P-STAIR is a feasible and acceptable treatment for reducing maternal PTSD symptoms, increasing positive parenting skills, and preventing maltreatment recidivism. Following P-STAIR, mothers had a 7-fold lower rate of new confirmed maltreatment reports (2.7%) compared to the FPS population in New York City (NYC) as a whole (18.6%). At the baseline assessment, all mothers met PTSD criteria and nearly all (85.8%) met criteria for comorbid depression. At the 90-day follow-up, 92.3% of mothers receiving P-STAIR no longer met diagnostic criteria for PTSD. Independent behavioral observations demonstrated a significant reduction of negative parenting behaviors and increased positive parenting behaviors. A randomized controlled trial (RCT) is now required to evaluate the efficacy of P-STAIR compared to supportive counseling (SC), an active treatment condition, in reducing child maltreatment recidivism. The RCT will further test maternal PTSD and depression symptom reduction and parenting skills improvement.

### Objectives {7}

- Aim 1: Compare P-STAIR to SC on change in maternal PTSD/depression.
  - H1: From baseline assessment to 6-month follow-up, P-STAIR will (a) be more effective in reducing maternal PTSD and depression symptoms; (b) have greater PTSD remission rates (scores<20 on the Clinician-Administered PTSD Scale for DSM-5 (CAPS-5)) [16]; and (c) have greater rates of no longer meeting PTSD criteria.
- Aim 2: Compare P-STAIR to SC with respect to parenting behaviors.
  - H2: P-STAIR will be more effective than SC in (a) decreasing observed negative parenting behaviors; and (b) increasing observed positive parenting behaviors, measured by raters using the standardized Parent-Child Interaction Therapy (PCIT) Dyadic Parent-Child Interaction Coding System (DPICS-IV) [17]; (c) P-STAIR will improve parenting attitudes more than SC.
- Aim 3: Compare P-STAIR to SC with respect to maltreatment recidivism.
  - H3a: P-STAIR will be more effective than SC in reducing new substantiated reports of maltreatment.
  - H3b: Reduced PTSD/depression symptoms and reduced negative parenting will mediate the effects of P-STAIR on maltreatment recidivism.

### Trial design {8}

**Fig. 1:**
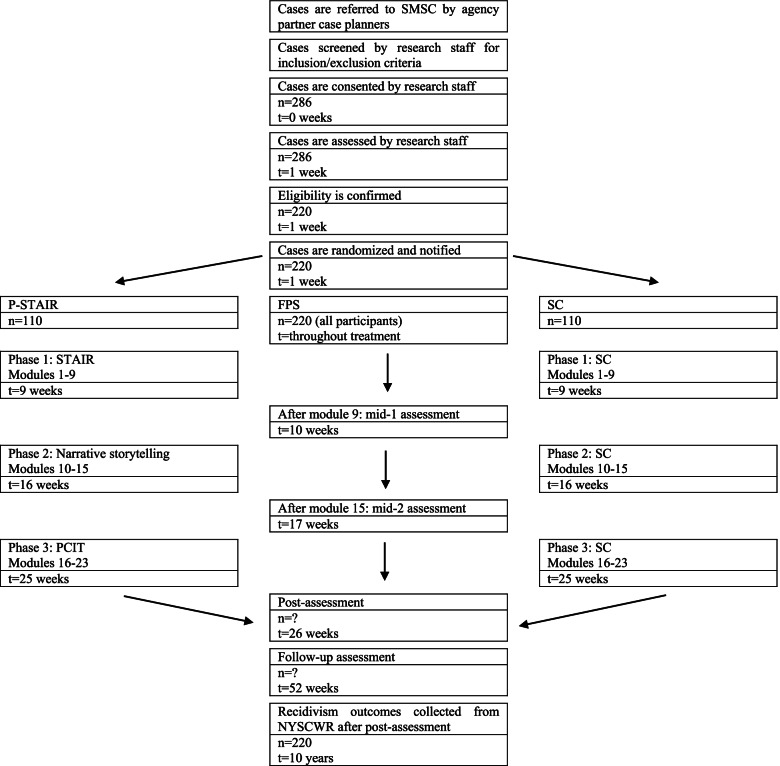
Study flow

**Fig. 2:**
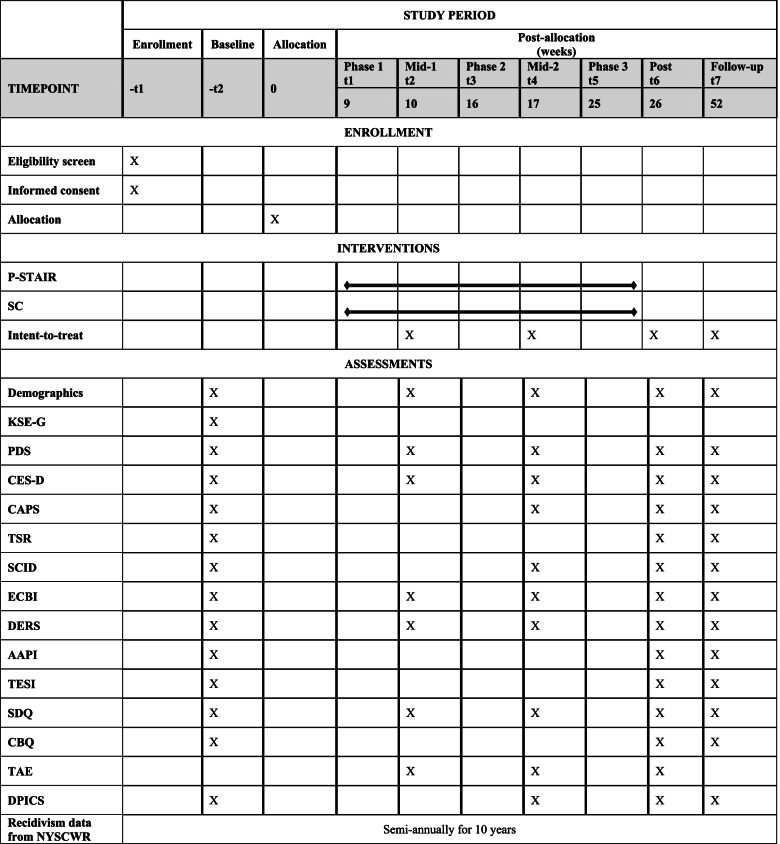
Schedule of enrollment, interventions, and assessments

The SMSC study is designed as a randomized controlled superiority trial with two treatment groups: P-STAIR and SC. We will enroll a total of 220 participants over 4 years: half (*N* = 110) randomly assigned to the P-STAIR condition and half (*N* = 110) to the supportive counseling condition. Each intervention is 23 sessions, and clinical assessments occur at pre-treatment (baseline), two in-treatment assessments (mid-assessment 1 after module 9 and mid-assessment 2 after module 15), post-treatment, and at a 6-month follow-up (see Figs. 1 and 2). All treatment sessions and assessments are recorded to monitor fidelity.

## Methods: participants, interventions, and outcomes

### Study setting {9}

All study activities will be conducted in New York City, NY, USA, at New York University (NYU) and our five preventive service agency partners: Association to Benefit Children (ABC), Children’s Aid Society (CA), Graham Windham (GW), Vibrant Emotional Health, and JCCA, formally known as Jewish Child Care Association.

### Eligibility criteria {10}

Inclusion criteria:

- Receiving child welfare preventive services
- Meeting DSM-5 diagnostic criteria for PTSD with or without co-occurring depression (CAPS-5)
- Having a child aged 1–8 who resides with the participant
- Being the legal guardian and having primary custody of the participating child
- Being able to read, write, and speak in English or Spanish

Exclusion criteria:

- Having suicidal ideation present in the past month prior to baseline or reports of a suicide attempt in the past year (Structured Clinical Interview for DSM-5 (SCID-5)) [18]
- Meeting DSM-5 criteria for substance or alcohol use disorder in the past month (SCID-5)
- Having symptoms or diagnosis of psychosis as defined by the DSM-5 in the past year (SCID-5)
- Having a different ability affecting communication, such as deafness
- Having an index child with a developmental condition that impedes functioning, e.g., autism spectrum condition, and/or a history of symptoms of psychosis during childhood
- Experiencing current intimate partner violence (IPV)/family violence or having a history of IPV/family violence
  - ° If there is a history of IPV/family violence and the relationship is no longer active, the relationship must have ended for at least 90 days with no intention of restarting
  - ° If there is a history of IPV/family violence, but the relationship is ongoing, there must not have been an event for at least 1 year

### Who will take informed consent {26a}

Research staff will meet clients at the agency, provide information about the research project and obtain consent from clients who are interested in participating before conducting a detailed baseline assessment to determine if the clients meet inclusion criteria. Research staff consenting participants are primarily study clinicians, but the clinical director, project manager, and research coordinator are approved and trained to consent when necessary. During the consenting process, research staff will read the consent form with the potential participant, offering explanations of each section and opportunities to ask questions at the end of each section. Research staff will inform potential participants that participation is completely voluntary, and it will not affect any preventive services or their relationship to Administration for Children’s Services (ACS), the child protective service agency in NYC. The consent form outlines that the participant will be randomly assigned to either P-STAIR or supportive counseling and details that research staff and preventive services case planners will communicate throughout treatment to enhance engagement and service delivery. During consent, research staff will review confidentiality and exceptions to confidentiality. Participation, regardless of randomization, is explained to the participant as adjunctive to the usual care provided through preventive services.

Following consent and the baseline assessment, the principal investigator, co-investigators, clinical director, and project manager will review the assessment, establish eligibility, and follow the random assignment procedures established. After randomization into P-STAIR or SC, participants will proceed to enrollment. Prior to beginning treatment sessions, participants will complete a 15-min dyadic play observation with the index child. Before the observation starts, the research staff facilitating the assessment will receive oral assent of the index child.

### Additional consent provisions for collection and use of participant data and biological specimens {26b}

Participants consent to the collection and use of data. In addition, consent forms state that individual identifiable data is not shared outside research staff. Use of identifiable information would require additional consents and written permission. The consent forms also outline limits to confidential information: mandated reporting or explicit approval from the participant. Participants are informed that data collected during treatment is not automatically deleted upon withdrawal. This trial does not involve collecting biological specimens.

## Interventions

### Explanation for choice of comparators {6b}

Supportive counseling (SC) is the comparison condition. As SC is an active treatment, the present study can be viewed as comparing two interventions equated for attention, with one targeting PTSD and associated mediators, while the other targets life and parenting problems without teaching specific skills to manage trauma and parenting. Although this is a higher bar for evaluating P-STAIR, the design is suited to mediation tests around trauma constructs.

### Intervention descriptions {11a}

The Safe Mothers, Safe Children study involves the comparison of P-STAIR versus supportive counseling. P-STAIR is an innovative combination of two evidence-based interventions: Skills Training in Affect and Interpersonal Regulation (STAIR) and PCIT [19–24]. STAIR, developed by Dr. Marylene Cloitre, is a two-phase treatment to improve emotion regulation and interpersonal skills prior to exposure treatment. STAIR is more effective than exposure alone and reduces dropouts for child abuse-related PTSD [25]. STAIR has been adapted to focus on the impact of PTSD (and related depression) on parenting. PCIT is a promising dyadic treatment for reducing recidivism in child welfare [26]. An abbreviated version of PCIT, including live coaching in dyadic sessions with the parent and index child, is used to focus on improving parenting skills [22, 26–30]. The P-STAIR treatment manual was developed collaboratively with Dr. Cloitre and co-investigators, Dr. Anthony Urquiza and Dr. Susan Timmer, of the UC-Davis PCIT Training Center. P-STAIR consists of 23 weekly, one-hour, treatment sessions divided into three phases: 1) STAIR; modules 1-9; 2) narrative storytelling; modules 10-15; and 3) Dyadic PCIT/applying parenting skills; modules 16-23. Homework is assigned after each module to practice parenting and emotional regulation skills used during sessions.

SC is an evidence-based, active treatment that controls for the “nonspecific” factors common to most psychotherapy [14, 31–40]. SC has been shown to be an acceptable and credible treatment in a number of RCTs involving trauma victims. Dr. Cloitre adapted the SMSC SC manual from the manual used by Dr. Edna Foa for sexual assault and trauma recovery [36]. The SC treatment is also 23 sessions. Session 1, similar to P-STAIR, will review the participant’s history, symptoms, and treatment rationale. Remaining sessions consist of participant-generated and directed discussions of life problems related to the participant’s trauma. Trauma-related skills training is excluded from SC, but non-trauma discussions of parenting problems is included, keeping with the focus of P-STAIR. SC clinicians play an unconditionally supportive role. Homework, consisting of keeping a diary of daily problems and problem-solving attempts, is offered but not assigned.

### Criteria for discontinuing or modifying allocated interventions {11b}

There will be no special criteria for modifying allocated intervention. Participation is voluntary and participants are able to discontinue or withdraw from treatment at any time. All participants, regardless of assignment, will complete all 23 sessions. Both treatment arms allow for non-protocol sessions to be conducted if clinically relevant for engagement, participation, or safety. Necessity is determined by the study clinician in collaboration with the clinical director, FPS case planner, and participant. Other modifications to treatment protocol include delaying sessions, e.g., because of travel, illness, appointments, school engagements, work, and technology issues.

### Adherence {11c}

To evaluate treatment adherence, all sessions and assessments are recorded (audio and video) with participant consent. Fidelity manuals have been developed for both treatment arms which include session objectives and adherence ratings. To measure adherence, two raters will be employed and trained on treatment protocols. Throughout study implementation, 20% of sessions/assessments will be randomly reviewed (stratifying for session number) and rated for manual adherence by an independent rater. To ensure reliability and guard against drift, a second independent rater will rate 10% of the selected sessions/assessments. Raters, the principal investigator, clinical director, and project manager will discuss reviews weekly.

Before beginning treatment with a participant, manual competency must be achieved. Competency will be defined as achieving 75% adherence over 23 sessions of training cases. If a study clinician fails to reach 75% adherence, they will complete additional supervised sessions with the clinical director until this criterion is achieved. Once treatment begins, all study clinicians receive ongoing weekly, one-hour, individual supervision and an hour and a half weekly group supervision with the clinical director. During supervision, the clinical director will provide bi-monthly feedback from fidelity ratings. If at any point, a clinician falls below 75% treatment fidelity, they will repeat training.

All dyadic observation coding is conducted at UC-Davis under the supervision of Dr. Urquiza and Dr. Timmer. PCIT adherence criteria have been established by UC-Davis. Coders will be trained directly by Dr. Urquiza and Dr. Timmer. These coders will evaluate dyadic observations preformed at pre-treatment (baseline), mid-treatment, post-treatment, and follow-up to determine positive and negative parenting ratings. Weekly reliability checks will occur to ensure a minimum of 85% reliability will be sustained by coders.

### Concomitant care {11d}

All enrolled participants are also receiving Family Preservation Services Usual Care (FPSUC), including case management. Mental health treatment outside participation in the study is permitted but tracked throughout study involvement using the Treatment Services Review. FPSUC will be accounted for in the analytic plan.

### Provisions for post-trial care {30}

Throughout treatment, all participants will receive any necessary referrals for additional mental health services. Study clinicians work directly with case planners to ensure continuity of care after withdrawal or treatment completion.

### Outcomes {12}

After a baseline assessment, symptom progress is measured at four additional timepoints for all participants: mid-assessment 1 after module 9, mid-assessment 2 after module 15, post-treatment, and 6-month follow-up. Primary outcomes for Aim 1 are the Center for Epidemiological Studies-Depression (CES-D) for depressive symptoms and the CAPS-5 for PTSD symptoms [16, 41]. Secondary PTSD and depression outcome measures include Post-traumatic Stress Diagnostic Scale for the DSM-5 (PDS-5) and Structured Clinical Interview for DSM-5 (SCID-5) [18, 42]. The Difficulties in Emotion Regulation Scale (DERS) is included as an exploratory measure [43].

The primary outcome for Aim 2 is DPICS-IV for measuring parenting skills [17]. Adult Adolescent Parenting Inventory-2.1 (AAPI-2.1) is used as a secondary outcome to assess parental attitudes towards children and child-rearing [44]. Other secondary outcomes for Aim 2 include child-focused measures: Children’s Behavior Questionnaire-Very Short Form (CBQ-VSF), Eyberg Child Behavior Inventory (ECBI), Strengths and Difficulties Questionnaire (SDQ), and Traumatic Events Screening Inventory-Parent Report Revised (TESI-PRR) [45–48].

The primary outcome for Aim 3 is maltreatment recidivism operationalized as new substantiated reports of child maltreatment and new removals to foster care. Treatment Services Review (TSR) will be used to statistically control for FPSUC [49]. Finally, the Treatment Acceptability and Expectations (TAE) scale will be used to evaluate the credibility and acceptability of treatments. The TAE was developed by Dr. Cloitre for use in the study.

### Participant timeline {13}

See Fig. 1 for a depiction of the timeline for participants.

### Sample size {14}

SMSC aims to enroll a total of 220 participants over the course of 4 years. Half (*N* = 110) will be randomly assigned to the P-STAIR treatment arm, and the other half (*N* = 110) will be randomly assigned to the supportive counseling treatment arm. In general, for PTSD, depression, and parenting measures, e.g., DPICS, our sample size has sufficient power to detect between condition effects of Cohen’s *d*=0.40 or greater. Norms in the field are to power studies to detect Cohen’s *d* of 0.50 or more; otherwise, effects are too small to be clinically meaningful. The vast majority of similar trials reporting target effect sizes were powered to detect medium (*d*=0.50) to large (*d*=0.80) minimum detectable effects [50–62].

### Recruitment {15}

Participants will be recruited from five collaborating preventive services agencies in New York City. The case planners at preventive agencies conduct trauma screenings as part of their usual care protocol and will offer a study flyer to clients who endorse any prior trauma exposure. This study flyer will have contact information for research staff. If the client is interested in the study, case planners will confirm the clients’ willingness to be contacted by the research staff for further follow-up and informed consent.

## Assignment of interventions: allocation

### Sequence generation {16a}

Following the baseline assessment and confirmation of eligibility, participants will be randomly assigned to either the experimental or control group with a 1:1 allocation per a computer-generated randomization schedule.

### Concealment mechanism {16b}

The randomization schedule will not be shared outside of the principal investigator, co-investigators, and project manager to ensure concealment. The randomization schedule is stored on a password-protected, secured web-based platform. The schedule is stored separately from randomization assignments to further ensure confidentiality. The sequence of assignments will not be released until the participant is enrolled, after baseline assessment and eligibility confirmation.

### Implementation {16c}

All participants who consented and met inclusion criteria will be randomized. Randomization will be relayed to the assigned study clinician by the project manager. The randomization schedule was prepared by one of the study’s co-investigators and sent to the project manager prior to study implementation. For each client randomized, the project manager will access the schedule, inform the assigned clinician, and record the assignment in a password-protected spreadsheet. Participants are informed of treatment assignments in their first session. The principal investigator will monitor randomization protocols monthly to safeguard that the allocation sequence is properly executed. The schedule cannot be edited by any staff. Thus, randomization is conducted without influence from research staff.

## Assignment of interventions: blinding

### Who will be blinded {17a}

In order to reduce risk of confirmation bias, research staff assessing participants are blinded to treatment condition. Assessments during and after study implementation are conducted by non-treatment clinicians. Due to the nature of the interventions and study design, neither participants nor treatment-administering staff will be blinded to allocation, but all discussions of assessment results after randomization allocation exclude treatment clinicians, fidelity raters, and coders. During group supervision, case-level information is not discussed.

Case planners and other agency staff are also blinded from treatment conditions. Participants are instructed not to share treatment arm designation or treatment-specific information with their case planners. Study clinicians and case planners will collaborate to enhance client engagement, but the focus of this collaboration will be on contact, not content (e.g., no direct discussion of the treatment plan, sessions completed, or anything that would jeopardize the randomization blinding). Assessment reports will be provided to case planners for each participant at the baseline, mid, and post-assessment with an overview of symptom progress and client engagement only. Assessment reports are prepared by a non-treating clinician and stored in a separate folder on a web-based platform. Folders are not accessible between clinicians.

### Procedure for unblinding if needed {17b}

Unblinding will only occur in exceptional circumstances in the case of serious adverse events (discussed below), when knowledge of treatment assignment is essential for the safety and further care of the participant.

## Data collection and management

### Plans for assessment and collection of outcomes {18a}

Data collection points are at pre-treatment (baseline assessment), two mid-point assessments, post-treatment, and at follow-up (see Fig. 2). All measures are unmodified. All Spanish translations created by the developers/publishers were used unless otherwise noted. Clinicians have been trained by the clinical director for uniform administration. Demographic data will be collected at all assessment timepoints, including date of birth for participant and index child, gender, ethnicity, insurance, employment status, socioeconomic level, marital status, current diagnosis(es), current medication, ACS allegation details, and referral source. Two proxy questions on motivation and stress level to account for selection bias have been created for use in the baseline assessment. The English-language adaption of the Social Desirability—Gamma Short Scale (KSE-G) is a six-item Likert scale used to gauge social desirability. KSE-G has demonstrated good construct validity and sufficient reliability [63]. The KSE-G is only administered at the baseline assessment. The Spanish-language version of the KSE-G was translated through the method of translation-back-translation by two bilingual (Spanish-English) SMSC staff [64, 65]. There are no official Spanish translations of this measure.

#### Primary outcomes

CAPS-5 is a 30-item structured clinical interview established by the U.S. Department of Veterans Affairs National Center for PTSD (NCPTSD). It yields a categorical measure of diagnosis and a severity score. Severity scores are calculated from 20 DSM-5 PTSD symptoms and range from 0 (“absent”) to 80 (“extreme”). CAPS-5 has established strong interrater and test-retest reliability for diagnosis and severity scores [16]. CAPS-5 will be used to assess change in PTSD diagnosis criteria and severity over treatment implementation (baseline assessment, mid-assessment 2, post-assessment, and follow-up assessment). CAPS-5 is also used at the baseline assessment to evaluate inclusion criteria.

There is no official NCPTSD translation of the CAPS-5. The Spanish-language version of CAPS-5 used was developed by Maria Jose Rendon at the University of Miami [66]. This version was selected because the cultural adaptation method highlights the sociocultural and linguistic nuances of complex symptomatology, diagnosis of trauma/PTSD, and the variety of Spanish dialects.

CES-D is a 20-item self-report measure of symptoms related to depression, e.g., sleep patterns, appetite changes, and feelings of isolation. Items are rated on a 3-point scale from 0 (“rarely or none of the time”) to 3 (“most or almost all of the time”). Scores range from 0 to 60. High scores signify more depressive symptoms. CES-D has demonstrated good sensitivity and specificity and high internal consistency [67, 68]. CES-D will be used to assess change in depression symptoms over treatment implementation. CES-D will be completed at all timepoints.

DPICS-IV examines the quality of parent-child social interaction in three 5-min situations: child-directed play, parent-directed play, and clean-up. Positive skills include praise, reflect, and describe, and negative skills include questions, commands, and criticisms [17]. Observations are coded by trained DPICS coders to produce total scores. DPICS has demonstrated interrater reliability, discriminative and convergent validity, and treatment sensitivity [69]. DPICS is administered following the baseline assessment, mid-assessment 2, post-assessment, and follow-up assessment during 15-min dyadic play observations. DPICS scores are used to track changes in positive and negative parenting over treatment implementation.

To assess maltreatment recidivism, data are collected through the New York State Child Welfare Registry (NYSCWR) semi-annually for ten years. Collected data include the number of out-of-home placements and new substantiated child welfare reports for both completers and non-completers who have consented into the study. Number of new foster care removals and new substantiated reports are located by unique NYSCWR identifiers collected from FPS at the time of consent.

#### Secondary outcomes

AAPI-2.1 is a self-report inventory that examines parenting behaviors. The measure is used to evaluate the risk of child maltreatment. Items are rated on a scale of 1 (“strongly agree”) to 5 (“strongly disagree”). AAPI-2.1 has five sub-constructs: expectations of children, parental empathy towards children's needs, use of corporal punishment, parent-child family roles, and children’s power and independence. Higher scores indicate lower risk of parental abuse/neglect [44]. Total scores have adequate validity. AAPI has strong correlations with other parent and child behavioral measures [70, 71]. AAPI will be used to monitor change in parental behaviors over treatment implementation at the baseline assessment, post-assessment, and follow-up assessment.

CBQ-VSF is a 36-item parent-report of child temperament. Items are rated on a 7-point scale from 1 (“extremely untrue of my child”) to 7 (“extremely true of my child”). The questionnaire has three sub-scales: surgency/extraversion, negative affectivity, and effortful control [45]. This measure has acceptable internal consistency and criterion validity [45, 72]. CBQ-VSF will be used to assess change over treatment implementation in the temperament of the index child at the baseline assessment, post-assessment, and follow-up assessment.

DERS is a 36-item self-report measure used to assess emotion regulation. Items are rated on a 5-point scale from 1 (“almost never [0-10%]”) to 5 (“almost always [91-100%]”). Examples include “I know exactly how I am feeling;” “When I’m upset, I feel out of control;” and “When I’m upset, it takes me a long time to feel better.” Higher scores represent a higher likelihood of difficulty with emotion regulation [65]. DERS has adequate construct and predictive validity and good test-retest reliability [43, 73]. DERS will be used to evaluate the level of emotion regulation skills during treatment implementation at all assessment timepoints.

ECBI is a 36-item parent-report measure designed to assess conduct issues in children ages 2-16. ECBI asks parents to identify how often a behavior is currently happening with their child on a 7-point scale from 1 (“never”) to 7 (“always”; intensity scores). Then, parents indicate whether or not a behavior is a problem through a “yes/no” response (problem scores) [46]. Higher total intensity scores indicate a higher frequency of behaviors. Higher total problem scores suggest more behaviors are a problem for the parent. This measure has acceptable test-retest and interrater reliability, internal consistency, and construct validity [74–78]. ECBI will be used throughout treatment to assess the change in behavior of the child participating in the parent-child dyadic play observations and the frequency of parent-identified problems.

PDS-5 is a 24-item self-report measure of PTSD symptoms over the last month. Items rate frequency and severity of symptoms on a 5-point scale from 0 (“not at all”) to 4 (“6 or more times a week/severe”). Higher scores signal more severe PTSD symptoms. A cutoff score of 28 indicates a likely PTSD diagnosis [42]. PDS-5 is a reliable and valid measure of PTSD symptomatology using DSM-5 criteria with excellent internal consistency and test-retest reliability and good convergent validity with other PTSD scales [42]. Together with CAPS-5, PDS-5 will be used to monitor changes in PTSD symptoms over treatment implementation. PDS-5 will be administered at all timepoints.

SDQ is a 25-item parent-report behavioral screening questionnaire that is comprised of five sub-scales: emotional symptoms, conduct problems, hyperactivity/inattention, peer relationship problems, and prosocial behavior. Items have the following responses: “not true,” “somewhat true,” and “certainly true” [47]. Higher scores denote a higher likelihood of emotional and/or behavioral difficulties. The reliability of the measure is satisfactory and criterion validity has been established [79, 80]. SDQ will be used to assess the change in behavior of the child participating in the parent-child dyadic play observations. SDQ is administered at all assessment timepoints.

SCID-5 is the gold standard for determining DSM-5 current diagnoses and psychiatric history [18]. The SCID-5 depression, alcohol and substance use, and psychosis modules will be used. Symptom scales of the SCID have been proven reliable and valid with good internal consistency, test-retest reliability, and concurrent and predictive validity [81]. SCID-5 will be used to track changes in depression, substance use, and symptoms of psychosis, as well as to evaluate inclusion/exclusion criteria. SCID-5 will be completed at baseline, mid-assessment 2, post-assessment, and follow-up assessment.

TAE is a 5-item scale that evaluates the credibility of and engagement in treatment. TAE is administered throughout treatment to assess the clinician-client therapeutic relationship and client engagement with treatment. This scale was developed by Dr. Cloitre to assess treatment engagement specific to this type of intervention. TAE is used at mid-assessment 1, mid-assessment 2, and post-assessment. The Spanish-language version of the TAE was translated through the method of translation-back-translation by two bilingual (Spanish-English) SMSC staff [64, 65]. There are no official Spanish translations of this measure.

TESI-PRR is a 24-item parent-report measure for preschool-age children used to assess the frequency and type of child exposure to traumatic events [48]. Earlier versions of the TESI-PRR, TESI-Parent Report, have demonstrated test-retest reliability [82]. A community adapted TESI has demonstrated validity for screening for adverse childhood experiences (ACEs) [83]. The TESI-PRR will be used throughout treatment to monitor trauma exposures of the index child prior to enrollment and changes while in treatment at baseline, post-assessment, and follow-up assessment.

TSR is an interview used to gather information about specific mental health services received outside of the study treatment, lifetime treatment history, and treatment received at the time of the baseline assessment. The interview details treatment type (individual vs. group), provider type (psychologist, psychiatrist, social worker), length (in years) and frequency of treatment, number of hospitalizations, and medications prescribed [49]. The TSR will be used throughout treatment to monitor mental health services prior to enrollment and changes while in treatment (baseline, post-assessment, and follow-up assessment).

### Plans to promote participant retention and complete follow-up {18b}

Multiple pathways promote participant retention, enable outreach, and reduce barriers to treatment engagement. Participants in both treatment arms will be provided compensation of $50 each for baseline assessment, post-treatment, and follow-up assessments and $30 for each of the four videotaped 15-min mother-child play interaction assessments (at baseline assessment, mid-treatment 2, post-treatment, and at follow-up). Participants will also be provided $10 in compensation for participating in each of the 23 regular treatment sessions and $30 for completing the two mid-assessments. Metro cards will be provided to cover transportation costs. Compensation helps to defray potential barriers to engagement, like childcare. Participants will only receive compensation for completed study activities, and compensation will not be contingent on study completion. Compensation is outlined during the consent process.

In addition to incentives, multiple avenues of contact with participants, including collecting support system contact information during informed consent, appointment reminders, and support for transportation, promote retention. Treatment is co-located in FPS agencies to reduce access barriers. In addition, in each treatment session, clinicians and participants review practical and emotional barriers to engagement.

Participants have the right to withdraw from treatment at any time. The principal investigator may also withdraw participants because of safety concerns or attendance non-adherence. Consistent with planned intent-to-treat analyses, participants who withdraw or drop out of treatment will continue to be assessed according to the study timeline. For participants who discontinue treatment, their rationale will be documented.

### Data management {19}

All study data will be entered weekly into SPSS by research staff trained by the project manager on entry protocols documented in the standard operating procedures for the study. Data will be monitored and supervised by the project manager, under the oversight of the principal investigator. Data will be audited bi-weekly through case summary reports. Data entry errors will be corrected, if applicable, and/or documented. Auditing reports will be distributed to the principal investigator for review. Original assessments will be stored in participant files on a secure web-based platform using only the study identification code. No names will be recorded in the assessment or database. Original assessments will be kept for 6 years after study completion, as outlined during informed consent.

### Confidentiality {27}

In order to track study referrals prior to the consent process, the principal investigator, clinical director, project manager, and research coordinator will have access to a password-protected referral log. Data from the study will be kept on a secure server. The materials for each participant will be identified by identification numbers only. No names will be put on any study materials, with the exception of consent and assent forms, which will be stored in a separate location from treatment and assessment files. The code linking participant identifiers to their research data will be stored in a separate password-protected file on the secure server and will only be accessible to principal investigator, clinical director, project manager, and research coordinator. In addition, all study materials, including session notes and assessments, are recorded through REDCap, which requires a virtual private network internet transmission to access. Audio and video recordings are stored in separate folders from other participant information and sorted by clinician. Any recordings viewed outside the study research staff, i.e., for DPICS coding, are de-identified through blurring all faces and identifiable marks and adjusting the voice pitch.

As one purpose of this study is to test a behavioral intervention to reduce child maltreatment recidivism, we will consult the child welfare database to obtain recidivism data. Specific consent forms from each participant allow access to these databases. Each participant’s unique child welfare identifier is obtained from FPS with consent; only this identifier is used to gain access to substantiated claims.

### Plans for collection, laboratory evaluation, and storage of biological specimens for genetic or molecular analysis in this trial/future use {33}

There will be no biological specimens collected in this study.

## Statistical methods

### Statistical methods for primary and secondary outcomes {20a}

For all analyses, we will evaluate for outliers and functional form misspecification. Estimation will use Huber-White robust estimation as implemented in Mplus or bootstrapping to accommodate non-normality and variance heterogeneity, as appropriate. Where possible, we will pursue sensitivity analyses that allow us to address bias due to measurement error [84]. Though we do not expect clustering to be an issue, we will be sensitive to clustering due to site and clinicians within sites, with adjustments using the clustering algorithms in Mplus. We will explicitly test for site differences in treatment effects. We will adjust for multiple contrasts using a Holm-modified Bonferroni method but recognize Bayesian caution against doing so and the need for sensitivity analyses both with and without them [85, 86]. For all multi-item scales, we will evaluate unidimensionality and composite reliability using confirmatory factor analysis.

Analysis of HYP. 1(a): This hypothesis will be tested in multiple ways. One approach will use a mixed effects model (MEM) defined by the following equation:

1 $${Y}_{it}={\beta}_0+{\beta}_1{I}_{i0}+{\beta}_2{I}_i^{P\hbox{-} STAIR}+{\beta}_3t+{\beta}_4t\ast {I}_i^{P\hbox{-} STAIR}+{\gamma}_1{I}_i^{site}+{\gamma}_2{I}_i^{MH}+{error}_{it}$$

where *Y*_*it*_ is the value of the symptom for *i*th subject at time *t*, *I*_*i*_^P-STAIR^ is an indicator for P-STAIR treatment having value 1 if the *i*th subject is assigned P-STAIR and 0 otherwise, *I*^site^ is a vector of indicators for agency site that the *i*th subject was recruited from, *I*^MH^ is an indicator whether or not the *i*th case was receiving mental health services, and error_*it*_ is the error term consisting of a random subject intercept plus random error. We also will approach the data using a more traditional between-condition single degree of freedom contrast for the relevant outcome at follow-up with the baseline assessment and other relevant control variables as covariates (i.e., an ANCOVA type model). This entails comparing a mediator or outcome at a given point in time (e.g., at post-test and at the mid-treatments) using the baseline measure as a covariate. Within-condition across-time contrasts of interest can be pursued using single degree of freedom contrasts in the mixed effect modeling (MEM) frame.

Analysis of HYP. 1(b): The facets of PTSD at the 6-month follow-up constitute binary outcomes. The hypothesis will be tested using logistic/probit regression (or another form of binary regression as dictated by the data) to model the binary variable with the treatment group and other potential confounders adjusted as covariates.

Analysis of HYP. 2(a,b,c): These hypotheses will be tested using the strategy described for Hyp. 1(a). For parenting attitudes, analyses will include total scores and subscale scores.

Analysis of HYP. 3a: We will use a Bayesian-based z-test to compare the proportion of families with substantiated maltreatment reports as well as the proportion of cases with children placed in foster care. An exact test will be used if these proportions are near 0. We will test condition differences using an appropriate binary regression model, for which treatment assignment, site, and other relevant covariates will be used as predictors. We will examine differences between P-STAIR and SC groups using a count outcome of the number of maltreatment reports 1 year after treatment. An appropriate count model (Poisson, negative binomial, and their ZIP counterparts) will be isolated and applied [87–89].

Analysis of HYP. 3b: Tests of mediation will evaluate the statistical significance of each path in the mediational chain of interest (via the joint significance test of MacKinnon) in a structural equation modeling framework [90]. The model will include paths from the mediators to the outcome, from the P-STAIR condition to the mediators/outcome, and relevant covariates. A causal path from PTSD symptoms to parenting behaviors (DPICS) will be included if our prior analyses suggest this is reasonable.

### Interim analyses {21b}

No interim analyses are anticipated.

### Methods for additional analyses (e.g. subgroup analyses) {20b}

No additional analyses are planned.

### Methods for protocol non-adherence or missing data {20c}

Primary analyses will be conducted using an intent-to-treat population. Additionally, Complier Average Causal Effect (CACE) and per-protocol populations analyses will be conducted to evaluate efficacy [91]. ITT analysis compares outcomes for all randomized individuals, estimating the effects of randomization to condition on outcomes. CACE identifies treatment individuals who complied with treatment protocols and compares their outcomes with control individuals who likely would have been treatment compliers had they been assigned to the treatment condition. Per-protocol analysis compares outcomes for treatment compliers to all control individuals. Each estimate is informative.

### Plans for access to full protocol, participant-level data, and statistical code {31c}

The full protocol, datasets analyzed during the current study, and statistical code are available from the corresponding author upon reasonable request.

## Oversight and monitoring

### Composition of coordinating centers and trial committees {5d}

The coordinating center of the study is the research staff at McSilver Institute for Poverty Policy and Research at New York University (NYU). The decision-making body of the study consists of the principal investigator (Michael Lindsey), the co-investigators (Kathrine Sullivan and James Jaccard), senior project staff (Kelly Ancharski and Mercedes Okosi). Together, the research team works in tandem with all other working parts of the study to maintain compliance, study deliverables, and treatment fidelity.

### Composition of the data monitoring committee: role and structure {21a}

A data safety and monitoring board (DSMB), consisting of a chair and two members, oversees the study. All members are independent of the RCT and have no financial, scientific, or other conflict of interest with the study and include experts in PTSD, clinical trial methodology, child welfare, and biostatistics. Under the leadership of the chair, Dr. Lisa Dixon, the DSMB will focus on the conduct and progress of the study with special attention to pooled safety and efficacy data. The DSMB will evaluate study conduct (accrual), safety (adverse events), data integrity (subject eligibility, protocol deviations), and the risk benefit ratio for trial participants. The DSMB will meet quarterly to conduct ongoing review of protocol compliance.

### Adverse event reporting and harms {22}

Several mechanisms are in place to monitor potentially adverse events that participants may experience while enrolled in the study, whether they are related to project participation or not. These events are classified as either reportable, adverse, or not harmful/expectable, as described below, and will be reported to the NYU IRB, DSMB, and NICHD, as appropriate. Per the NYU IRB, a reportable event is an unanticipated problem involving risks to participants or others (“Unanticipated Problem”) and any event or information that (1) was unforeseen and (2) indicates that the research procedures caused harm to participants or others or indicates that participants or others are at increased risk of harm. Some examples of reportable events for the present study might be (1) emotional breakdown requiring psychiatric intervention as a result of study participation; (2) suicidal threat or behavior as a result of study participation; namely, the serious threat or attempt to inflict serious bodily harm to oneself that may result in death; (3) serious violent threats or behaviors as a result of study participation including any threats, ideations or attempts to seriously injure or kill another person; or (4) experiences of IPV/family violence as a result of study participation. Reportable (unanticipated; related to study participation; harmful) events will be reported to NYU IRB, DSMB, and NICHD within 24 h and additional details of event and actions taken within 72 h. All DSMB findings are communicated to the IRB(s) and NICHD. Any additional requests for information by NYU IRB, DSMB, and/or NICHD will be responded to accordingly.

Adverse but not “reportable” (anticipated in the consent form or unanticipated; may or may not be associated with study) events will be documented on the protocol deviations log, including whether it appears to be related to study participation. These events will be reported in aggregate or summary form to NYU IRB annually, DSMB at each scheduled meeting, and NICHD during the progress report. Clinical referrals will be provided at the time of event, as needed. Not harmful/expectable (largely anticipated and not harmful) events are not reported to NYU IRB, DSMB, or NICHD, but clinical referrals will be provided at the time of event.

### Frequency and plans for auditing trial conduct {23}

A semi-annual internal audit will be conducted by the DSMB to evaluate protocol compliance including consent, inclusion/exclusion criteria, proper data storage, efficacy, and participant safety. Bi-weekly data audits will be administrated by the research team.

### Plans for communicating important protocol amendments to relevant parties {25}

Each year, the study will be renewed through NYU IRB. Any interim changes to the IRB-approved materials or protocol require additional IRB approval. Annually, research staff will update the New York State Office of Children and Family Services (OCFS) and ACS on project status, including information about proposals sent to NYU IRB and outside organizations and any protocol modifications approved by NYU IRB. Other reporting will be to ClinicalTrials.gov and NICHD. Necessary changes to the ClinicalTrials.gov registration will be updated by research staff as modifications are approved by NYU IRB. Protocol amendments will be communicated to NICHD during progress reports.

### Dissemination plans {31a}

SMSC is committed to the open and timely dissemination of research outcomes. In compliance with NYU and NIH policy, this clinical trial has been registered with ClinicalTrials.gov. The coordination team is responsible for aggregate results and adverse event reporting at the conclusion of the study. Study results will be submitted not later than 1 year after the primary completion date. All study investigators are aware of and agree to abide by the principles for sharing research resources, as described by NIH. The data generated in this RCT will be presented at national or international conferences and published in a timely fashion. All final peer-reviewed manuscripts that arise from this proposal will be submitted to the digital archive PubMed Central. In addition, working with our collaborators at ACS, we will ensure broad dissemination of our treatment procedures (should results warrant) to the child welfare community.

## Discussion

This is the first RCT to evaluate P-STAIR. If demonstrated efficacious, P-STAIR will reduce maltreatment recidivism among high-risk child welfare involved mothers, ameliorating lives of children and families, and reducing mental health stigma. The results from this study are likely to have considerable policy and funding impacts regarding preventive services and evidence-based care mandates in line with the Family First Prevention Services Act of 2018 [92].

In this first study of P-STAIR, the target population is mothers and their children, but for future evaluations of the intervention, SMSC aims to implement P-STAIR for other populations, especially fathers involved in the child welfare system. P-STAIR also has the potential to be used in broader settings beyond preventative services. SMSC believes, based on the flexibility and adaptability of the treatment in the pilot, P-STAIR can be implemented via different modalities, e.g., virtually and in-person. In the COVID-19 pandemic, the pilot successfully transitioned to virtual treatment. This also opens the door for future studies that examine virtual vs. in-person efficacy of the intervention. While testing is needed to determine reliability and validity, establishing different iterations of P-STAIR will expand the utility and accessibility of mental health care for parents with PTSD.

## Trial status

This is protocol version 1.0, dated July 26, 2021. Recruitment began on May 26, 2021. Primary completion is anticipated on June 20, 2025, with final study completion anticipated on April 30, 2026.

## Data Availability

Only the principal investigator, co-investigators, project manager, and research coordinator have access to full datasets. De-identified datasets will be accessible to consultants and other research staff. All datasets are stored on a password-protected, secured web-based platform.

## Abbreviations

AAPI-2.1: Adult Adolescent Parenting Inventory-2.1
ABC: Association to Benefit Children
CES-D: Center for Epidemiological Studies-Depression
CBQ-VSF: Children’s Behavior Questionnaire-Very Short Form
CA: Children’s Aid Society
CAPS-5: Clinician-Administered PTSD Scale for DSM-5
DERS: Difficulties in Emotion Regulation Scale
DPICS-IV: Dyadic Parent-Child Interaction Coding System-IV
ECBI: Eyberg Child Behavior Inventory
GW: Graham Windham
IRB: Institutional Review Board
NICHD: National Institute of Child Health and Human Development
ACS: New York City Administration for Children's Services
OCFS: New York State Office of Children and Family Services
NYU: New York University
PCIT: Parent-Child Interaction Therapy
P-STAIR: Parenting-STAIR
PDS-5: Post-traumatic Stress Diagnostic Scale for the DSM-5
SMSC: Safe Mothers, Safe Children
STAIR: Skills Training in Affective and Interpersonal Regulation
SDQ: Strengths and Difficulties Questionnaire
SCID-5: Structured Clinical Interview for DSM-5
SC: Supportive counseling
TESI-PRR: Traumatic Events Screening Inventory-Parent Report Revised
TAE: Treatment Acceptability and Expectations
TSR: Treatment Services Review

## References

[1] U.S. Department of Health & Human Services, Administration for Children and Families, Administration on Children, Youth and Families, Children’s Bureau. Child maltreatment 2018. https://www.acf.hhs.gov/cb/data-research/child-maltreatment. Accessed 25 Jan 2022.

[2] Wang CT, Holton J, America PCA (2007). Total estimated cost of child abuse and neglect in the United States.

[3] Gilbert R, Widom CS, Browne K, Fergusson D, Webb E, Janson S (2009). Burden and consequences of child maltreatment in high-income countries. Lancet.

[4] Felitti VJ, Anda RF, Lanius RA, Vermetten E, Pain C (2010). The relationship of adverse childhood experiences to adult medical disease, psychiatric disorders and sexual behavior: Implications for healthcare. The impact of early life trauma on health and disease: The hidden epidemic.

[5] Nemeroff CB (2016). Paradise lost: the neurobiological and clinical consequences of child abuse and neglect. Neuron.

[6] Fang X, Brown DS, Florence CS, Mercy JA (2012). The economic burden of child maltreatment in the United States and implications for prevention. Child Abuse Negl.

[7] Macmillan HL, Wathen CN, Barlow J, Fergusson DM, Leventhal JM, Taussig HN (2009). Interventions to prevent child maltreatment and associated impairment. Lancet.

[8] Chaffin M, Hecht D, Bard D, Silovsky JF, Beasley WH (2012). A statewide trial of the SafeCare home-based services model with parents in Child Protective Services. Pediatrics.

[9] Conron KJ, Beardslee W, Koenen KC, Buka SL, Gortmaker SL (2009). A longitudinal study of maternal depression and child maltreatment in a national sample of families investigated by child protective services. Arch Pediatr Adolesc Med.

[10] Famularo R, Fenton T, Kinscherff R, Ayoub C, Barnum R (1994). Maternal and child posttraumatic stress disorder in cases of child maltreatment. Child Abuse Negl.

[11] De Bellis MD, Broussard ER, Herring DJ, Wexler S, Moritz G, Benitez JG (2001). Psychiatric co-morbidity in caregivers and children involved in maltreatment: a pilot research study with policy implications. Child Abuse Negl.

[12] Chemtob CM, Gudiño OG, Laraque D (2013). Maternal posttraumatic stress disorder and depression in pediatric primary care: association with child maltreatment and frequency of child exposure to traumatic events. JAMA Pediatr.

[13] Hicks LM, Dayton CJ (2019). Mindfulness and trauma symptoms predict child abuse potential in risk-exposed, men and women during pregnancy. Child Abuse Negl.

[14] Chemtob CM, Griffing S, Tullberg E, Roberts E, Ellis P (2011). Screening for trauma exposure, and posttraumatic stress disorder and depression symptoms among mothers receiving child welfare preventive services. Child Welfare.

[15] Scott JC, Matt GE, Wrocklage KM, Crnich C, Jordan J, Southwick SM (2015). A quantitative meta-analysis of neurocognitive functioning in posttraumatic stress disorder. Psychol Bull.

[16] Weathers FW, Bovin MJ, Lee DJ, Sloan DM, Schnurr PP, Kaloupek DG (2018). The Clinician-Administered PTSD Scale for DSM-5 (CAPS-5): development and initial psychometric evaluation in military veterans. Psychol Assess.

[17] Eyberg SM, Nelson MM, Ginn NC, Bhuiyan N, Boggs SR (2013). Dyadic parent-child interaction coding system: comprehensive manual for research and training.

[18] First MB, Williams JBW, Karg RS, Spitzer RL (2015). Structured clinical interview for DSM-5: Research Version (SCID-5 for DSM-5, Research Version; SCID-5-RV).

[19] Cloitre M, Koenen KC, Cohen LR, Han H (2002). Skills training in affective and interpersonal regulation followed by exposure: a phase-based treatment for PTSD related to childhood abuse. J Consult Clin Psychol.

[20] Jain S, Ortigo K, Gimeno J, Baldor DA, Weiss BJ, Cloitre M (2020). A randomized controlled trial of brief Skills Training in Affective and Interpersonal Regulation (STAIR) for veterans in primary care. J Trauma Stress.

[21] MacIntosh HB, Cloitre M, Kortis K, Peck A, Weiss BJ (2018). Implementation and evaluation of the Skills Training in Affective and Interpersonal Regulation (STAIR) in a community setting in the context of childhood sexual abuse. Res Soc Work Pract.

[22] Thomas R, Zimmer-Gembeck MJ (2011). Accumulating evidence for parent-child interaction therapy in the prevention of child maltreatment. Child Dev.

[23] Thomas R, Abell B, Webb HJ, Avdagic E, Zimmer-Gembeck MJ (2017). Parent-child interaction therapy: a meta-analysis. Pediatrics.

[24] Timmer SG, Urquiza AJ, Zebell NM, McGrath JM (2005). Parent-child interaction therapy: application to maltreating parent-child dyads. Child Abuse Negl.

[25] Cloitre M, Stovall-McClough KC, Nooner K, Zorbas P, Cherry S, Jackson CL (2010). Treatment for PTSD related to childhood abuse: a randomized controlled trial. Am J Psychiatry.

[26] Chaffin M, Funderburk B, Bard D, Valle LA, Gurwitch R (2011). A combined motivation and parent-child interaction therapy package reduces child welfare recidivism in a randomized dismantling field trial. J Consult Clin Psychol.

[27] Kennedy SC, Kim JS, Tripodi SJ, Brown SM, Gowdy G (2016). Does parent–child interaction therapy reduce future physical abuse? A meta-analysis. Res Soc Work Pract.

[28] Hakman M, Chaffin M, Funderburk B, Silovsky JF (2009). Change trajectories for parent-child interaction sequences during parent-child interaction therapy for child physical abuse. Child Abuse Negl.

[29] Nixon RD, Sweeney L, Erickson DB, Touyz SW (2004). Parent-child interaction therapy: one- and two-year follow-up of standard and abbreviated treatments for oppositional preschoolers. J Abnorm Child Psychol.

[30] Timmer SG, Thompson D, Culver MA, Urquiza AJ, Altenhofen S (2012). Mothers’ physical abusiveness in a context of violence: effects on the mother-child relationship. Dev Psychopathol.

[31] Heimberg RG, Dodge CS, Hope DA, Kennedy CR, Zollo LJ, Becker RE (1990). Cognitive behavioral group treatment for social phobia: comparison with a credible placebo control. Cogn Ther Res.

[32] Heimberg RG, Liebowitz MR, Hope DA, Schneier FR, Holt CS, Welkowitz LA (1998). Cognitive behavioral group therapy vs phenelzine therapy for social phobia: 12-week outcome. Arch Gen Psychiatry.

[33] Borkovec TD, Mathews AM, Chambers A, Ebrahimi S, Lytle R, Nelson R (1987). The effects of relaxation training with cognitive or nondirective therapy and the role of relaxation-induced anxiety in the treatment of generalized anxiety. J Consult Clin Psychol.

[34] Borkovec TD, Mathews AM (1988). Treatment of nonphobic anxiety disorders: a comparison of nondirective, cognitive, and coping desensitization therapy. J Consult Clin Psychol.

[35] Craske MG, Maidenberg E, Bystritsky A (1995). Brief cognitive-behavioral versus nondirective therapy for panic disorder. J Behav Ther Exp Psychiatry.

[36] Foa EB, Rothbaum BO, Riggs DS, Murdock TB (1991). Treatment of posttraumatic stress disorder in rape victims: a comparison between cognitive-behavioral procedures and counseling. J Consult Clin Psychol.

[37] Shear MK, Pilkonis PA, Cloitre M, Leon AC (1994). Cognitive behavioral treatment compared with nonprescriptive treatment of panic disorder. Arch Gen Psychiatry.

[38] Teusch L, Böhme H, Gastpar M (1997). The benefit of an insight-oriented and experiential approach on panic and agoraphobia symptoms. Results of a controlled comparison of client-centered therapy alone and in combination with behavioral exposure. Psychother Psychosom.

[39] Bryant RA, Sackville T, Dang ST, Moulds M, Guthrie R (1999). Treating acute stress disorder: an evaluation of cognitive behavior therapy and supportive counseling techniques. Am J Psychiatry.

[40] Bryant RA, Harvey AG, Dang ST, Sackville T, Basten C (1998). Treatment of acute stress disorder: a comparison of cognitive-behavioral therapy and supportive counseling. J Consult Clin Psychol.

[41] Radloff LS (1977). The CES-D scale: a self-report depression scale for research in the general population. Appl Psychol Meas.

[42] Foa EB, McLean CP, Zang Y, Zhong J, Powers MB, Kauffman BY (2016). Psychometric properties of the Posttraumatic Diagnostic Scale for DSM-5 (PDS-5). Psychol Assess.

[43] Gratz KL, Roemer L (2008). Multidimensional assessment of emotion regulation and dysregulation: development, factor structure, and initial validation of the difficulties in emotion regulation scale. J Psychopathol Behav Assess.

[44] AAPI-2.1. https://assessingparenting.com/assessment/aapi. Accessed 25 Jan 2022.

[45] Putnam SP, Rothbart MK (2006). Development of short and very short forms of the Children’s Behavior Questionnaire. J Pers Assess.

[46] Eyberg SM, Ross AW (1978). Assessment of child behavior problems: the validation of a new inventory. J Clin Child Psychol.

[47] Goodman R (1997). The strengths and difficulties questionnaire: a research note. J Child Psychol Psychiatry.

[48] Ghosh-Ippen C, Ford J, Racusin R, Acker M, Bosquet K, Rogers C (2002). Traumatic events screening inventory – parent report revised.

[49] McLellan AT, Alterman AI, Cacciola J, Metzger D, O’Brien CP (1992). A new measure of substance abuse treatment. Initial studies of the treatment services review. J Nerv Ment Dis.

[50] Cohen JA, Mannarino AP, Iyengar S (2011). Community treatment of posttraumatic stress disorder for children exposed to intimate partner violence: a randomized controlled trial. Arch Pediatr Adolesc Med.

[51] Spence J, Titov N, Dear BF, Johnston L, Solley K, Lorian C (2011). Randomized controlled trial of Internet-delivered cognitive behavioral therapy for posttraumatic stress disorder. Depress Anxiety.

[52] Litz BT, Salters-Pedneault K, Steenkamp MM, Hermos JA, Bryant RA, Otto MW (2012). A randomized placebo-controlled trial of D-cycloserine and exposure therapy for posttraumatic stress disorder. J Psychiatr Res.

[53] Forbes D, Lloyd D, Nixon RD, Elliott P, Varker T, Perry D (2012). A multisite randomized controlled effectiveness trial of cognitive processing therapy for military-related posttraumatic stress disorder. J Anxiety Disord.

[54] Schnurr PP, Friedman MJ, Engel CC, Foa EB, Shea MT, Chow BK (2007). Cognitive behavioral therapy for posttraumatic stress disorder in women: a randomized controlled trial. JAMA.

[55] Mueser KT, Rosenberg SD, Xie H, Jankowski MK, Bolton EE, Lu W (2008). A randomized controlled trial of cognitive-behavioral treatment for posttraumatic stress disorder in severe mental illness. J Consult Clin Psychol.

[56] Ehlers A, Hackmann A, Grey N, Wild J, Liness S, Albert I (2014). A randomized controlled trial of 7-day intensive and standard weekly cognitive therapy for PTSD and emotion-focused supportive therapy. Am J Psychiatry.

[57] Sannibale C, Teesson M, Creamer M, Sitharthan T, Bryant RA, Sutherland K (2013). Randomized controlled trial of cognitive behaviour therapy for comorbid post-traumatic stress disorder and alcohol use disorders. Addiction.

[58] Talbot LS, Maguen S, Metzler TJ, Schmitz M, McCaslin SE, Richards A (2014). Cognitive behavioral therapy for insomnia in posttraumatic stress disorder: a randomized controlled trial. Sleep.

[59] Surís A, Link-Malcolm J, Chard K, Ahn C, North C (2013). A randomized clinical trial of cognitive processing therapy for veterans with PTSD related to military sexual trauma. J Trauma Stress.

[60] Johnson DM, Zlotnick C, Perez S (2011). Cognitive behavioral treatment of PTSD in residents of battered women’s shelters: results of a randomized clinical trial. J Consult Clin Psychol.

[61] Foa EB, McLean CP, Capaldi S, Rosenfield D (2013). Prolonged exposure vs supportive counseling for sexual abuse-related PTSD in adolescent girls: a randomized clinical trial. JAMA.

[62] Resick PA, Wachen JS, Mintz J, Young-McCaughan S, Roache JD, Borah AM (2015). A randomized clinical trial of group cognitive processing therapy compared with group present-centered therapy for PTSD among active duty military personnel. J Consult Clin Psychol.

[63] Nießen D, Partsch MV, Kemper CJ, Rammstedt B. An English-language adaptation of the social desirability–gamma short scale (KSE-G). Meas Instrum Soc Sci. 2019;1(1). 10.1186/s42409-018-0005-1.

[64] APA dictionary of psychology. https://dictionary.apa.org/. Accessed 25 Jan 2022.

[65] Tyupa S (2011). A theoretical framework for back-translation as a quality assessment tool. New Voices Transl Stud.

[66] Rendon MJ (2015). The cultural adaptation of the Clinician-Administered PTSD Scale for Spanish-speaking Latinos with limited English proficiency in the United States [dissertation on the Internet].

[67] Lewinsohn PM, Seeley JR, Roberts RE, Allen NB (1997). Center for Epidemiologic Studies Depression Scale (CES-D) as a screening instrument for depression among community-residing older adults. Psychol Aging.

[68] Carleton RN, Thibodeau MA, Teale MJ, Welch PG, Abrams MP, Robinson T (2013). The center for epidemiologic studies depression scale: a review with a theoretical and empirical examination of item content and factor structure. PLoS One.

[69] Nelson MM, Olsen B, Niec L (2018). Dyadic parent–child interaction coding system (DPICS): an adaptable measure of parent and child behavior during dyadic interactions. Handbook of parent-child interaction therapy.

[70] Lawson MA, Alameda-Lawson T, Byrnes E (2017). Analyzing the validity of the Adult-Adolescent Parenting Inventory for low-income populations. Res Soc Work Pract.

[71] Conners NA, Whiteside-Mansell L, Deere D, Ledet T, Edwards MC (2006). Measuring the potential for child maltreatment: the reliability and validity of the Adult Adolescent Parenting Inventory-2. Child Abuse Negl.

[72] de la Osa N, Granero R, Penelo E, Domènech JM, Ezpeleta L (2014). The short and very short forms of the Children’s Behavior Questionnaire in a community sample of preschoolers. Assessment.

[73] Hallion LS, Steinman SA, Tolin DF, Diefenbach GJ (2018). Psychometric properties of the Difficulties in Emotion Regulation Scale (DERS) and its short forms in adults with emotional disorders. Front Psychol.

[74] Funderburk BW, Eyberg SM, Rich BA, Behar L (2003). Further psychometric evaluation of The Eyberg and Behar rating scales for parents and teachers of preschoolers. Early Educ Dev.

[75] Eisenstadt TH, McElreath LH, Eyberg S, Bodiford McNeil C (1994). Interparent agreement on the Eyberg Child Behavior Inventory. Child Fam Behav Ther.

[76] Bearss KE, Eyberg S (1998). A test of the parenting alliance theory. Early Educ Dev.

[77] Bor W, Sanders MR (2004). Correlates of self-reported coercive parenting of preschool-aged children at high risk for the development of conduct problems. Aust N Z J Psychiatry.

[78] Burns GL, Patterson DR (2000). Factor structure of the Eyberg Child Behavior Inventory: a parent rating scale of oppositional defiant behavior toward adults, inattentive behavior, and conduct problem behavior. J Clin Child Psychol.

[79] Stone LL, Otten R, Engels RC, Vermulst AA, Janssens JM (2010). Psychometric properties of the parent and teacher versions of the strengths and difficulties questionnaire for 4- to 12-year-olds: a review. Clin Child Fam Psychol Rev.

[80] Goodman R (2001). Psychometric properties of the strengths and difficulties questionnaire. J Am Acad Child Adolesc Psychiatry.

[81] Shankman SA, Funkhouser CJ, Klein DN, Davila J, Lerner D, Hee D (2018). Reliability and validity of severity dimensions of psychopathology assessed using the Structured Clinical Interview for DSM-5 (SCID). Int J Methods Psychiatr Res.

[82] Stover CS, Berkowitz S (2005). Assessing violence exposure and trauma symptoms in young children: a critical review of measures. J Trauma Stress.

[83] Choi KR, McCreary M, Ford JD, Rahmanian Koushkaki S, Kenan KN (2019). Validation of the traumatic events screening inventory for ACEs. Pediatrics.

[84] Oberski DL, Satorra A (2013). Measurement error models with uncertainty about the error variance. Struct Equ Model.

[85] Dienes Z (2011). Bayesian versus orthodox statistics: which side are you on?. Perspect Psychol Sci.

[86] O’Keefe DJ (2003). Colloquy: should familywise alpha be adjusted?. Human Comm Res.

[87] Lambert D (1992). Zero-inflated Poisson regression with an application to defects in manufacturing. Technometrics.

[88] Hall DB (2000). Zero-inflated Poisson and binomial regression with random effects: a case study. Biometrics.

[89] Yau KK, Lee AH (2001). Zero-inflated Poisson regression with random effects to evaluate an occupational injury prevention programme. Stat Med.

[90] Jaccard J, Bo A (2018). Prevention science and child/youth development: randomized explanatory trials for integrating theory, method, and analysis in program evaluation. J Soc Soc Work Res.

[91] Jo B, Asparouhov T, Muthén BO, Ialongo NS, Brown CH (2008). Cluster randomized trials with treatment noncompliance. Psychol Methods.

[92] H.R.1892 - 115th Congress (2017-2018): Bipartisan Budget Act of 2018, H.R.1892, 115th Cong. 2018. https://www.congress.gov/bill/115th-congress/house-bill/1892.

